# The hidden costs of automation: does robot adoption affect children’s mental health?

**DOI:** 10.3389/fpsyg.2025.1643849

**Published:** 2025-10-09

**Authors:** Yueqing Zou, Jiaxin Wang, Liang Wang

**Affiliations:** ^1^Department of Business Administration, Zhejiang Institute of Administration, Hangzhou, China; ^2^Zhejiang Province “Eight-Eight Strategy” Innovation Development Institute, Zhejiang Institute of Administration, Hangzhou, China; ^3^School of Economics, Shanghai University of Finance and Economics, Shanghai, China; ^4^Business School, Xiangtan University, Xiangtan, China

**Keywords:** child mental health, robots, academic burden, living standards, parent–child interactions

## Abstract

**Introduction:**

Industrial automation is profoundly transforming the labor market, yet it may also impose hidden costs beyond economic outcomes. In particular, heightened labor market competition caused by robot adoption may create intergenerational costs, such as adverse effects on children’s mental health.

**Methods:**

This study combines survey data from the 2012-2020 China Family Panel Studies with robot data from the International Federation of Robotics to investigate the impact of automation on the mental health of Chinese children. To address endogeneity concerns, we construct an instrumental variable for domestic robot adoption using U.S. robot data and employ a two-stage least squares (2SLS) approach.

**Results:**

The findings reveal that a one-standard deviation increase in robot adoption (0.414 robots per thousand workers) increases the likelihood of severe depression among Chinese children by 0.75 percentage points. These adverse effects are largely mediated by heightened academic pressure and reduced positive parent-child interactions. Furthermore, the effects are stronger among girls and children living in urban areas.

**Discussion:**

This study advances understanding of the broader social implications of automation. It highlights the often-overlooked psychological costs of automation, demonstrating that its effects extend beyond the current workforce to future generations.

## 1 Introduction

Economies worldwide are undergoing rapid industrial automation. According to the International Federation of Robotics, the total number of operational robots globally more than doubled between 2011 and 2019. During this period, China, as a developing economy, experienced an even sharper increase, with the number of operational robots rising tenfold. A substantial body of literature has examined the effects of robot adoption on the labor market, including its impacts on employment (Acemoglu and Restrepo, 2020), wages (Acemoglu and Restrepo, 2020), and wage inequality (Lankisch et al., 2019; Ge and Zhou, 2020). Additionally, a growing body of research explores how workers and households respond to these labor market disruptions, such as changes in marriage patterns (Giuntella et al., 2022; Anelli et al., 2024), fertility rates (Giuntella et al., 2022; Matysiak et al., 2023; Anelli et al., 2024), and physical and mental health outcomes (Gihleb et al., 2022; Abeliansky et al., 2024). Industrial automation may also entail hidden costs, namely unintended, indirect, and less visible consequences that extend beyond immediate labor market outcomes. In particular, heightened labor market competition caused by robot adoption may create intergenerational costs, such as adverse effects on children’s mental health. Despite its importance, this dimension of adjustment at the child level has received limited attention in the existing literature.

Moreover, while existing research has extensively examined the effects of technological advancements in advanced economies, the impact on emerging markets and developing economies remains relatively unexplored. In the context of China, the adoption of robotics could have profound implications for children’s mental health for several reasons. First, China leads the world in manufacturing employment and has become the largest market for robotic applications (Gan et al., 2023). Second, although the country is deeply involved in labor-intensive manufacturing sectors, labor rights protections remain insufficient (Yu, 2008), potentially exacerbating the vulnerabilities of workers. Third, longstanding cultural norms in China, including parents’ reliance on children for old-age support and the deeply ingrained belief in education as a pathway to social and economic mobility, may amplify the transmission of labor market shocks caused by automation to children. These factors create a unique context where the ripple effects of technological change on younger generations are likely to be significant and warrant closer examination.

This paper seeks to address these gaps in the literature by examining the impact of exposure to industrial robots on children’s mental health in China. Two key labor market effects of robotics adoption may pose risks to children’s well-being: the widening skill wage gap (Acemoglu and Restrepo, 2020) and a decline in overall household income (Giuntella et al., 2022). On one hand, robots primarily displace low-skilled workers, leading to income shocks for these individuals, while exerting a much smaller negative impact—or even creating a skill premium—on high-skilled workers (Lankisch et al., 2019; Acemoglu and Restrepo, 2020). The skill-biased nature of automation, along with the resulting social inequality, may increase parental anxiety about their children’s future labor market prospects. This anxiety often manifests in the imposition of heavier academic burdens on children (Giuntella et al., 2022; Chen et al., 2023), which in turn may contribute to higher rates of depression among children (Jiang et al., 2021). On the other hand, households affected by negative income shocks may experience a decline in living standards. Financial stress could lead to reduced parental attention and fewer positive parent–child interactions (Brody et al., 1994), further compromising children’s mental health (McLoyd, 1990; Lauritzen and Sivertsen, 2012; Baird et al., 2013). As depression can severely impair young people’s social, educational, and occupational functioning (Thapar et al., 2022), it is essential to quantify the causal effect of robotics adoption on children’s mental health and identify the primary mechanisms driving this impact.

This paper investigates the impact of robot adoption on children’s mental health using survey data from the China Family Panel Studies (CFPS). The city-year level robot penetration rate is constructed using a shift-share approach, following the methodology of Acemoglu and Restrepo (2020). A key challenge to identification lies in the potential omitted variable bias, where both children’s mental health and robot adoption may be influenced by external factors. To address this endogeneity issue, we use foreign robot adoption as an instrumental variable for domestic robot application. The instrumental variable (IV) estimation results indicate that robot adoption significantly raises the likelihood of severe depression among children. These findings remain robust when alternative measures of mental health are used or when variations in the construction of robot adoption rates are considered. Furthermore, we account for a comprehensive set of potential confounding factors, including advancements in internet access, levels of foreign direct investment, and the intensity of educational competition in each city. These robustness checks strengthen the validity of our conclusions.

This paper further examines the mechanisms through which robot adoption impacts children’s mental health. The findings reveal that robot adoption significantly increases children’s academic burden, a factor closely linked to adolescent psychological issues (Jiang et al., 2021). Additionally, we explore whether adverse family income shocks contribute to the mental health effects and find that, while robot adoption does not significantly affect children’s living standards, it substantially reduces positive parent–child interactions. This reduction exacerbates children’s stress responses and negatively impacts their mental health (McLoyd, 1990). Our analysis concludes that increased academic pressure and diminished positive parent–child interactions are the primary drivers of the observed rise in severe depression among children. Further heterogeneity analysis indicates that these effects are particularly pronounced among girls and children living in urban areas, highlighting the nuanced impact of automation across different demographic groups.

This paper makes three key contributions to the existing literature. First, it extends the growing body of research on the impact of robots on individual well-being. While prior studies have primarily focused on how robots affect the labor market and adults’ well-being—exploring areas such as marriage (Giuntella et al., 2022; Anelli et al., 2024), fertility (Giuntella et al., 2022; Matysiak et al., 2023; Anelli et al., 2024), and physical and mental health (Gihleb et al., 2022; Abeliansky et al., 2024)—the potential effects on children have received little attention. This paper fills this gap by investigating the impact of robot adoption on children’s mental health and identifying the mechanisms through which these effects occur. By doing so, it broadens the understanding of the social implications of automation, particularly for younger, more vulnerable populations.

Secondly, this study introduces a novel influencing factor on children’s mental health: technological progress. Existing literature has primarily examined the determinants of children’s mental health at the individual and macro levels. Individual and family-level factors include parental divorce (Strohschein, 2005), parental migration (Wu et al., 2015), childhood maltreatment (Negriff, 2020), family socioeconomic status (Pryor et al., 2019), digital media use (Shutzman and Gershy, 2023), and social deprivation (Orben et al., 2020). At the macro level, research has highlighted influences such as green space (Engemann et al., 2019), digital technology (Hollis et al., 2020), and the COVID-19 pandemic (Newlove-Delgado et al., 2021).

Children’s mental health has profound implications, incurring costs not only for families but also for society at large. Poor mental health in childhood affects human capital accumulation (Currie and Stabile, 2006) and can lead to adverse outcomes such as increased involvement in crime (Anderson et al., 2015). By emphasizing the role of technological progress, this paper enriches the understanding of factors influencing children’s mental health and underscores the broader societal consequences of automation.

Thirdly, this study contributes to the literature by examining the effects of industrial robot exposure in an emerging economy. Existing research on the impact of robots on labor markets and individual well-being has predominantly focused on developed countries (Acemoglu and Restrepo, 2020, 2022; Gihleb et al., 2022; Matysiak et al., 2023; Anelli et al., 2024). However, as noted earlier, households and individuals in China may respond to robots differently compared to those in developed nations, due to distinct cultural, economic, and institutional factors. Understanding the hidden mental health costs of automation on children in China provides valuable insights into the broader social implications of technological progress in emerging markets. These findings can help policymakers worldwide design targeted strategies to mitigate mental health challenges and support a smoother transition to an automated economy, particularly in contexts with unique economic and social dynamics.

**Figure 1:**
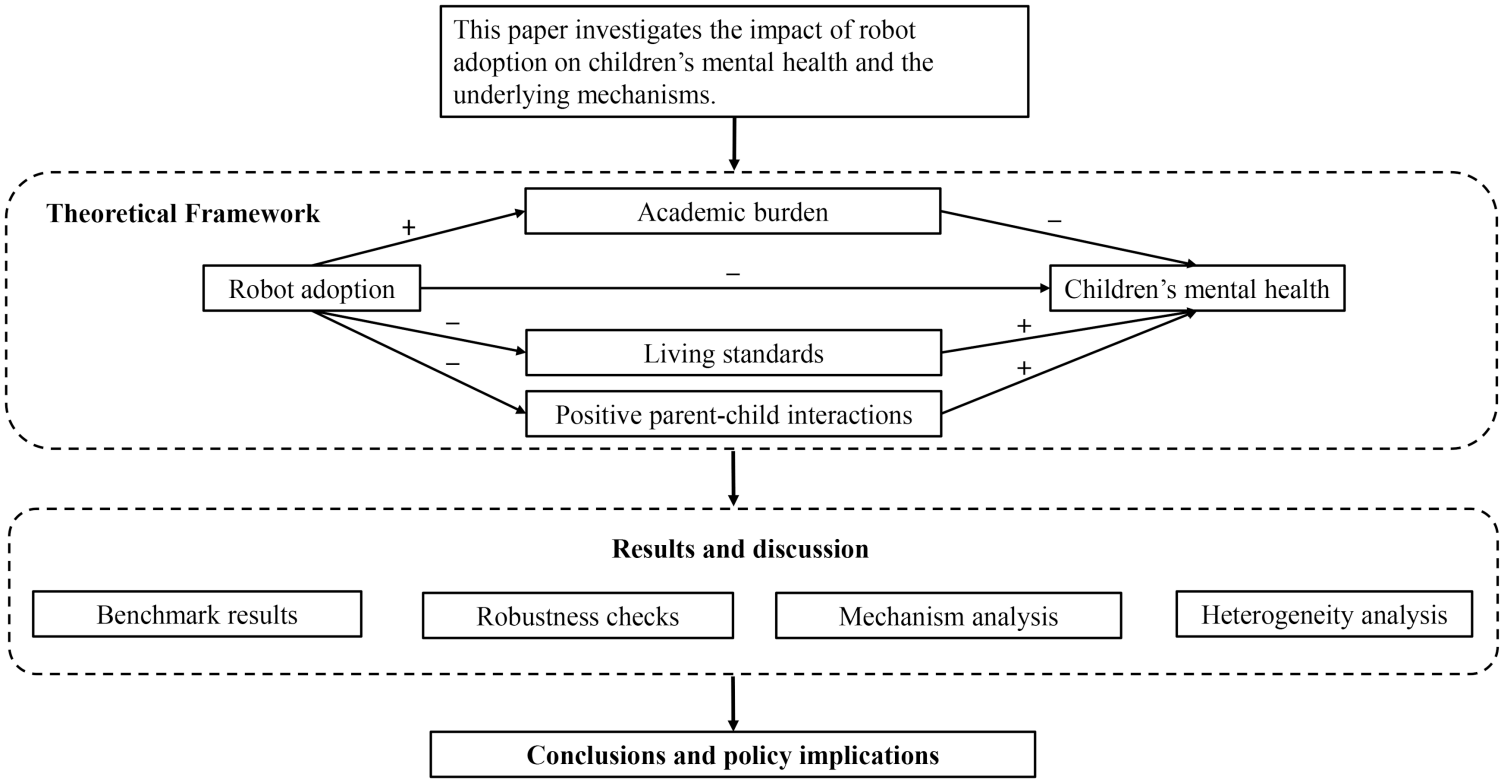
Theoretical framework and empirical process.

This study’s theoretical framework and empirical process are illustrated in Figure 1. The remainder of the paper is structured as follows: Section 2 outlines the methodology employed in the study. Section 3 details the empirical strategy used to analyze the data. Section 4 presents the research findings and provides a discussion of the results. Finally, Section 5 concludes the paper.

## 2 Method

### 2.1 Participants

We utilize data from the China Family Panel Studies (CFPS) 2012–2020. The analysis of restricted city-level data in this article was conducted in the restricted data room of the Center for Chinese Social Science Survey at Peking University. Since large-scale robot implementation in China began after 2010, we include survey data from 2012 onwards, which ensures sufficient variability in the independent variable by lagging the robot data by one period in the regression model. The CFPS is a national longitudinal survey conducted by the Institute of Social Science Survey (ISSS) at Peking University. It collects data at individual, family, and community levels, using a stratified, multi-stage sampling strategy to ensure the sample represents 95% of the total population of China (Xie and Hu, 2014). The sample covers compulsory education stage children aged 10 to 15, and each child is matched with their parents. After removing missing values and outliers, the final sample comprises 6,423 observations of children residing in 116 cities.

### 2.2 Measures

Our dependent variable is whether the child is severely depressed. The CFPS 2012, 2016, 2018, and 2020 employ the 20-item questionnaire from the Center for Epidemiological Studies-Depression Scale (CES-D), while the 2014 data utilize the 6-item Kessler Psychological Distress Scale (K6). Both scales are internationally validated, conceptually similar, and widely recognized measures of depressive symptoms (Rosenquist et al., 2011; Mewton et al., 2016; Tran et al., 2019). Combining CES-D and K6 allows us to maximize the use of all available CFPS survey waves and to provide a more comprehensive longitudinal perspective, a strategy also adopted by several recent studies (Wu et al., 2023; Zhu et al., 2025). Following the methodology of Zhu et al. (2025) and the CFPS technical report, the scores of negatively worded questions are reversed to ensure that higher scores consistently indicate a greater severity of psychological depression. Then, the total score is calculated by summing all question scores. However, we acknowledge potential limitations of combining two different scales, as differences in length and item content may introduce measurement heterogeneity. To mitigate this concern, following classic works such as Radloff (1991) and Prochaska et al. (2012), we set the cutoff value for severe depression in the CES-D questionnaire at 28 and in the K6 questionnaire at 13. Severe depression means necessitating mental health treatment and causing impairments in functioning, which is important for informing clinical interventions and health policy (Prochaska et al., 2012).

In order to obtain the key explanatory variable robot adoption, we use data from the International Federation of Robots (IFR). The IFR reports industrial robot data for approximately 100 countries from 1993 to 2022, covering more than 90% of the world robotics market. Accordingly, it stands as the leading authoritative robot statistics worldwide (Wang et al., 2022). Unfortunately, the IFR data on robots are available only at the country-year-industry level, but we require robot data specific to the city where each child resides. To convert robot use intensity from the industry level to the city level, China’s industry classification is matched with the IFR’s robot stock industry classifications, and industrial robot operations are grouped into 19 industries (j = 1,…,19). Following Acemoglu and Restrepo (2020), the paper employs a shift-share design in constructing a Bartik measure of robot penetration rate on the city level, see Equation (1):


(1) $$\text{Robot}s_{\mathrm{ct}}=\sum_{j}L_{\mathrm{jc},2005}\frac{\text{Robo}t_{j,t}}{\text{Emplo}y_{j,2005}}$$

where c , j , and t denote city, industry, and year, respectively. $\text{Robo}t_{j,t}$ represents the stock of robots in China in industry j in year t , where j is one of the 19 industries, namely, automobile, other vehicles (shipbuilding and aerospace), plastic and chemicals, metal products, machinery, food and beverages, basic metals, electronic products, non-metallic mineral products, wood processing, textile, paper and printing, other manufacturing industries, mining and quarrying, education and research, agriculture, forestry and fishing, utilities (electricity, gas, water supply), construction, and service. Given China’s extensive adoption of robots since 2010, the base year is set at 2005. $\text{Emplo}y_{j,2005}$ is the country’s total employment of industry j in 2005, and $L_{\mathrm{jc},2005}$ represents the base year employment share of industry *j* in city *c*. Employment data are either sourced from the WORLDKLEMS database or computed based on the 2005 1% National Population Sample Survey. The mean robot penetration rate stands at 0.206 robots per thousand workers, with a standard deviation of 0.414.

In estimation, we include a set of child and family demographic features that may influence children’s depression as controls, namely the child’s gender (1 = male, 0 = female), age, hukou status (1 = rural hukou, 0 = urban hukou), and number of siblings; and the parents’ age, years of education (illiterate or semi-literate = 0, primary school = 6, junior high school = 9, senior high school = 12, 3-year college = 15, 4-year college = 16, master’s degree = 19, doctoral degree = 21), and hukou status (1 = rural hukou, 0 = urban hukou).

An array of city characteristics are also included as control variables, namely (1) economic development level (LnGDP), obtained by taking the natural logarithm of GDP, (2) income level, measured as the logarithm of the city’s average wage of employees (in yuan), (3) cost of living, proxied by the logarithm of average house prices (in yuan), (4) environmental quality, measured as the logarithm of the annual average PM2.5 concentration in the city, and (5) educational resources, represented by the ratio of the number of students to the number of teachers in the nine-year compulsory education stage. Regarding the data source, the house prices are sourced from the China Real Estate Information database, the data for PM2.5 concentrations are provided by the Center for International Earth Science Information Network (CIESIN), and other city characteristics are obtained from the “China City Statistical Yearbook.”

The summary statistics of the sample are presented in Table 1. The sample covers 6,423 matched child–parent observations residing in 116 cities. According to the CFPS definition of adolescents, the sample covers 10 to 15-year-old adolescents who had sufficient cognitive ability to complete self-reported questionnaires. The children are on average 12.5 years old and relatively balanced in both genders. In the data, 2.2% of children are severely depressed. The figure is comparable to research focusing on Chinese children and adolescents (Xu et al., 2018), warranting the validity of the data. The statistics for other individual characteristics in the sample are also consistent with those that use child data from the CFPS, such as Wang and Yang (2022).

**Table 1:** Summary statistics.

Variables	Mean	SD	Min	Max
Panel A: dependent variable				
Severely depressed	0.022	0.146	0.000	1.000
Panel B: independent variable				
Robots	0.206	0.414	0.002	3.530
Panel C: instrument variable				
Robots_IV	1.056	1.447	0.04^a^	9.58
Panel D: individual characteristics				
Child’s gender	0.528	0.499	0.000	1.000
Child’s age	12.501	1.706	10.000	15.000
Child’s hukou status (1 = rural; 0 = urban)	0.784	0.412	0.000	1.000
Number of siblings	0.677	0.834	0.000	7.000
Father’s age	41.213	5.089	28.000	60.000
Father’s years of education	8.507	3.701	0.000	19.000
Father’s hukou status (1 = rural; 0 = urban)	0.778	0.415	0.000	1.000
Mother’s age	39.370	5.027	24.000	58.000
Mother’s years of education	7.277	4.252	0.000	22.000
Mother’s hukou status (1 = rural; 0 = urban)	0.795	0.404	0.000	1.000
Panel E: city characteristics				
LnGDP	16.782	0.966	14.34	19.67
Income level	10.858	0.350	9.45	12.03
Cost of living	8.555	0.464	7.65	10.45
Environmental quality	3.799	0.339	3.00	4.601
Educational resources	14.595	2.512	8.30	23.97

The sample covers 6,423 matched child–parent observations residing in 116 cities.

a Based on the distribution of extreme values in the user-submitted external variables, the CFPS project team applied fuzzification to these values in the table.

### 2.3 Empirical strategy

To identify the impact of robots on children’s mental health, this paper estimates Equation (2):


(2) $$\begin{array}{l} \text{Severely}\_\text{depresse}d_{\mathrm{ict}}=\beta_{0}+\beta_{1}\text{Robot}s_{c,t-1}\\ +\beta_{2}X_{\mathrm{ict}}+\beta_{3}Z_{c,t-1}+\delta_{c}+\varphi_{t}+u_{\mathrm{ict}} \end{array}$$

where the subscripts *i*, *c*, and *t* represent the child, the located city, and the survey year. The dependent variable $\text{Severely}\_\text{depresse}d_{\mathrm{ict}}$ is a dummy variable indicating whether the child is severely depressed in year *t*. Recognizing that the impact of robots on children’s mental health may take additional time, we use the one-period lagged natural logarithm of the robot penetration rate in city *c* as the independent variable, denoted as $\text{Robot}s_{c,t-1}$ . $X_{\mathrm{ict}}$ stands for individual characteristics, and $Z_{c,t-1}$ represents city characteristics lagged by one period. Moreover, the regression controls for city fixed effects $\delta_{c}$ and year fixed effects $\varphi_{t}$ to account for city-specific and time differences in child depression. The standard errors are clustered at the city level to account for potential correlations across individuals in the same city. $\beta_{1}$ is the parameter of interest, capturing the average effect of robots on the likelihood of a child being severely depressed.

The main challenge to our identification is the omitted variable bias. A city with more extreme climates may tend to use more robots, while having children with systematically worse mental health conditions. To address the potential endogeneity issue, we follow Acemoglu and Restrepo (2020) in using robot adoption of foreign countries to construct an instrumental variable for domestic robot adoption, as shown in Equation (3):


(3) $$\text{Robo}t_{\mathrm{ct}}^{\mathrm{US}}=\sum_{j}L_{\mathrm{jc},2005}\frac{\text{Robo}t_{j,t}^{\mathrm{US}}}{\text{Emplo}y_{j,2005}^{\mathrm{US}}}$$

where $\text{Robo}t_{j,t}^{\mathrm{US}}$ and $\text{Emplo}y_{j,2005}^{\mathrm{US}}$ are, respectively, the stock of robots in industry j in year t , and the total employment in industry *j* in 2005 in the United States. $L_{\mathrm{jc},2005}$ indicates the proportion of employment in industry *j* relative to the total employment in city *c* in 2005 in China.

The US industrial robot data has been widely used in the literature for constructing IV for robot adoption in China (Giuntella et al., 2022; Liu et al., 2024). The validity of this practice is outlined as follows: Firstly, although the US outperforms China in robotics technology, the developmental trajectories between the two countries exhibit a certain degree of similarity (Zhu et al., 2023), thus affirming the relevance between the instrumental variable and robot exposure in Chinese cities. Secondly, automation in the US has a limited direct impact on the mental health of Chinese children, thereby ensuring the exogeneity of the instrumental variable. A potential concern is that global supply chains may transmit shocks from US automation to Chinese industries. For instance, increased robot adoption in the US might reduce demand for Chinese exports or shift the production strategies of multinational corporations, which could in turn influence Chinese labor markets and even children’s mental health. However, such channels are unlikely to be quantitatively important. While US robot adoption may marginally affect specific sectors in China through trade, the overall macroeconomic impact is limited due to China’s diversified export structure (Jiao et al., 2024). Additionally, the period under study coincides with China’s rapid domestic industrial automation and the expansion of the internal market. These domestic forces dominate employment and social outcomes, making indirect effects through global supply chains quantitatively minor.

The validity of the identification is further illustrated by testing for the exogeneity of the shift-share instrumental variable. According to Borusyak et al. (2022), it would suffice to prove the exogeneity of the shock from robot adoption. We follow Borusyak et al. (2022) and conduct a balance test on the city level to demonstrate that the foreign adoption of industrial robots is uncorrelated to the pre-trend demographic and socioeconomic conditions in China. The results presented in Table 2 indicate that the instrumental variable derived from foreign robot data is not correlated to city-level pre-trend characteristics such as the population share of women, average age, share of college-educated population, average income, average working hours, unemployment rate or undergraduate admission rates in each city in China. This suggests that the robot adoption shock can be considered as randomly assigned with respect to China’s regional demographic features, economic conditions or competition in higher education. The results provide support for the assumption that the instrumental variable satisfies the exogeneity condition.

**Table 2:** Balance test for the exogeneity of the instrumental variable.

Variables	(1)	(2)	(3)	(4)	(5)	(6)	(7)
	Share of women	Average age	Share of college-educated population	Ln (average income)	Average working hours	Unemployment rate	Undergraduate admission rate
Robots_IV	−0.005	−0.317	0.009	−0.067	−1.896	0.012	0.057
	(0.013)	(0.698)	(0.017)	(0.055)	(2.056)	(0.054)	(0.037)
Observations	580	580	580	580	580	580	580
R-squared	0.323	0.607	0.854	0.946	0.558	0.558	0.436

This table presents the regional balance test for the exogeneity of the instrumental variable. All columns include controls for the interaction of fixed effects between the year and the employment share of each industry in the city during the base period. Standard errors are calculated following Borusyak et al. (2022). ***, **, and * mean significance at the 1, 5, and 10% levels, respectively.

## 3 Results and discussion

### 3.1 Benchmark results

Columns 1–2 of Table 3 present the results of OLS regressions. Column 1 controls for city and year fixed effects. Column 2 additionally controls for individual and city characteristics. The OLS estimates suggest a significantly positive relationship between robot adoption and children being severely depressed.

**Table 3:** Baseline results.

	Severely depressed (1)	Severely depressed (2)	Robots (3)	Severely depressed (4)	Robots (5)	Severely depressed (6)
			2SLS		2SLS	
	OLS	OLS	First stage	Second stage	First stage	Second stage
Robots	0.012**	0.015**		0.016***		0.018***
	(0.006)	(0.007)		(0.006)		(0.007)
Robots_IV			0.576***		0.575***	
			(0.011)		(0.012)	
Individual characteristics	No	Yes	No	No	Yes	Yes
City characteristics	No	Yes	No	No	Yes	Yes
City FE	Yes	Yes	Yes	Yes	Yes	Yes
Year FE	Yes	Yes	Yes	Yes	Yes	Yes
N	6,423	6,423	6,423	6,423	6,423	6,423
R^2^	0.024	0.028	0.944	0.000	0.946	0.004
Kleibergen-Paap F statistic				2990.153		2384.449

Standard errors in parentheses are clustered at the city level. ***, **, and * mean significance at the 1, 5, and 10% levels, respectively.

Columns 4 and 6 report the two-stage least squares estimates with specifications of controls identical to those of Columns 1 and 2, and Columns 3 and 5 present the first-stage results for the two regressions, respectively. Both Kleibergen-Paap F statistics exceed 10, confirming the relevance of the instrumental variable. Specifically, based on the 2SLS estimates in Column 6, a one standard deviation increase in robot adoption (0.414 robots per thousand workers) significantly increases the probability of children being severely depressed by 0.75 percentage points.

In understanding the salience of the result, we note that China’s robot stock has experienced a rapid growth in recent years. From 2011 to 2019, China’s overall stock of robots on average increased by 0.144 standard deviation per year. Note that 2.7% of children are severely depressed in the 2020 sample. Based on the regression estimates, we can illustrate the potential impact of robot exposure during this specific period: the increase in robot adoption raised children’s risk of severe depression by 0.144*0.75*8 = 0.86 percentage points, accounting for 0.0086/0.027 = 31.85% of the children’s severe depression rate in 2020. This should be understood as an illustrative calculation based on the specific period of 2011–2019 and the 2020 prevalence rate. Nevertheless, the magnitude signifies a stark impact.

### 3.2 Robustness checks

Tables 4, 5 show the results of a battery of robustness checks. Firstly, our results are robust under a series of alternative specifications in Table 4. In Column 1 of Table 4, we use the robot data from the US, Netherlands, France, South Korea, and Germany—countries ahead of China in the advancement of automation, whose distribution of robots across industries is positively correlated with China—to construct an alternative instrumental variable. Column 2 changes the base year to 2010 for constructing the independent variable and instrumental variable. The industrial structures in 2010 are more up-to-date, and may hence better capture the shock from robots experienced by the city. Column 3 replaces the dependent variable with the child’s standardized depression score. A higher score indicates greater depression in the child. Furthermore, Column 4 uses children’s self-reported mental pressure ranging from 1 to 5, as an alternative measure of mental conditions, and the results show a consistent effect of robots in worsening the child’s mental conditions. Recognizing that industrial robots capture only one dimension of automation, we follow Nepal et al. (2025) and employ the number of AI-related patents as a proxy for urban AI development. AI-related patents are identified in the China Intellectual Property Office (CIPO) database based on specific International Patent Classification (IPC) codes. We substitute the independent variable with urban AI development and use the same instrumental variables as Nepal et al. (2025). The results are reported in Column 5. To mitigate potential errors caused by combining two different scales, CES-D and K6, to measure depression, we exclude the CFPS 2014 sample and rely solely on the CES-D questionnaire to determine whether a child is severely depressed. The corresponding results are reported in Column 6. Column 7 includes individual fixed effects in addition to the baseline specification to account for any individual-specific characteristics that might also be correlated with robot exposure, such as attitudes towards robot application. Besides, since we are using a shift-share identification strategy, the spatial correlation may occur across regions with similar sectoral composition. However, such correlations are not accounted for by the inference procedures (Adao et al., 2019). Column 8 applies the inference procedures proposed by Adao et al. (2019), which account for cross-region correlation in residuals within shift-share designs. The results are all largely similar.

**Table 4:** Robustness checks with alternative specifications.

Variables	Severely depressed	Severely depressed	Depression level	Pressure	Severely depressed	Severely depressed	Severely depressed	Severely depressed
	(1)	(2)	(3)	(4)	(5)	(6)	(7)	(8)
Robots	0.015**	0.012***	0.124*	0.433**		0.016***	0.056***	0.018***
	(0.006)	(0.004)	(0.071)	(0.197)		(0.006)	(0.019)	(0.006)
AI development					0.012***			
					(0.004)			
Individual characteristics	Yes	Yes	Yes	Yes	Yes	Yes	Yes	Yes
City characteristics	Yes	Yes	Yes	Yes	Yes	Yes	Yes	Yes
City fixed effects	Yes	Yes	Yes	Yes	Yes	Yes		Yes
Year fixed effects	Yes	Yes	Yes	Yes	Yes	Yes	Yes	Yes
Individual fixed effects							Yes	
N	6,423	6,423	6,423	4,286	6,423	4,828	4,228	6,423
R^2^	0.004	0.005	0.005	0.012	0.004	0.006	0.009	0.004
K-P F statistic	181.791	132.055	2384.449	411.883	100.035	1479.587	139.429	2384.449

Each column represents an independent 2SLS regression. Standard errors in parentheses are clustered at the city level. ***, **, and * mean significance at the 1, 5, and 10% levels, respectively.

**Table 5:** Robustness checks accounting for other confounding factors.

	Severely depressed								
	(1)	(2)	(3)	(4)	(5)	(6)	(7)	(8)	(9)
Robots	0.015**	0.012*	0.020**	0.019***	0.020***	0.027**	0.018**	0.018**	0.016**
	(0.007)	(0.007)	(0.008)	(0.007)	(0.006)	(0.013)	(0.007)	(0.007)	(0.006)
Individual characteristics	Yes	Yes	Yes	Yes	Yes	Yes	Yes	Yes	Yes
City characteristics	Yes	Yes	Yes	Yes	Yes	Yes	Yes	Yes	Yes
City fixed effects	Yes	Yes	Yes	Yes	Yes	Yes	Yes	Yes	Yes
Year fixed effects	Yes	Yes	Yes	Yes	Yes	Yes	Yes	Yes	Yes
N	6,423	5,587	6,423	6,423	6,423	5,456	6,423	6,423	6,423
R^2^	0.005	0.005	0.004	0.004	0.005	0.005	0.004	0.004	0.005
K-P F statistic	2828.126	716.737	884.900	2703.660	2369.500	529.895	2743.382	1913.701	2765.785

Each column represents an independent 2SLS regression. Standard errors in parentheses are clustered at the city level. ***, **, and * mean significance at the 1, 5, and 10% levels, respectively.

Additionally, we control for the effect of a series of factors that were changing concurrently with the application of robots in the city in Table 5. Column 1 of Table 5 controls for the internet penetration rate to eliminate the impact of rising information technology development on the results, ensuring that the observed effects are specifically attributable to robot exposure rather than general advancements in digital infrastructure. Besides, the offshoring of industries away from China due to the rising labor cost may be correlated with robot adoption, and it could affect job opportunities and academic pressure on children, which would bias the estimates upward. This concern is addressed by controlling for the city’s foreign direct investment in Column 2. A linear trend for the share of the largest industry (by employment) in the city is added in Column 3 to account for the dynamics of employment structure due to robot adoption. Column 4 incorporates both the child and elderly dependency ratios to control for the effects of family structure. Column 5 includes the strength of clan culture to account for local cultural influences. Following Huang et al. (2022), clan culture is measured by the number of regional genealogies relative to the regional population. This measure is further interacted with a time dummy variable to capture temporal variations. In Column 6, to exclude the effect of the COVID-19 outbreak in 2020, the sample is restricted to the CFPS 2012–2018 data.

Moreover, the admission rate of the college entrance examination has gradually increased with the expansion of China’s higher education in recent years, and government investment in education has also changed over time. These factors may correlate with the increasing use of robots and affect children’s academic burden and mental health, thereby potentially biasing the regression results. To address this concern, we collect data on the undergraduate admission rate and the first-tier college admission rate in each province from the Education Examinations Authority, and include them as controls, respectively, in Columns 7 and 8. Column 9 adds the ratio of regional education expenditure to total fiscal expenditure to account for variation in local education investment. The results remain largely consistent with these additional controls. Overall, the robustness of our results to various checks verifies the validity of our causal interpretation.

## 4 Mechanism analysis

Though seemingly immune from the direct impact of the labor market shocks caused by robots, children are still potentially affected through various ways. Previous studies have identified two primary labor market effects of robots: widening the skill wage gap (Acemoglu and Restrepo, 2020) and reducing overall household income (Giuntella et al., 2022). Both factors could potentially affect children’s mental health. Firstly, robots primarily cause displacement and income shocks for low-skilled workers, while having a much smaller negative impact or even raising the skill premium for high-skilled workers (Lankisch et al., 2019; Acemoglu and Restrepo, 2020). This skill-biased impact of robots on labor markets may heighten parental anxiety about their children’s future labor market prospects, which is often expressed through the imposition of heavier academic burdens on children (Giuntella et al., 2022; Chen et al., 2023). This may subsequently worsen children’s mental health (Jiang et al., 2021). Secondly, robot exposure reduces labor force participation, employment, and hourly wages of workers, causing families to experience negative income shocks (Giuntella et al., 2022). This may reduce the living standard for children. Furthermore, the negative income shock could cause parents to care less about their children’s lives and reduce positive parent–child interactions (Brody et al., 1994). In either case, children in the household may suffer from mental health problems (McLoyd, 1990; Lauritzen and Sivertsen, 2012; Baird et al., 2013).

### 4.1 Academic burden

This section explores the role of academic burden in mediating the impact of robots on child mental health. In light of this conjecture, we look into various indicators of children’s academic burden.

Columns 1 to 3 of Table 6 test the effect of robot adoption on the child’s studying time, whether or not the child attends tutorial classes, and the hours spent taking tutoring classes per week. The results indicate that robots increase both the amount of time children spend studying and the likelihood and duration of their attendance at tutoring classes. Columns 4 to 6 further study whether robots affect children’s studying attitude, using the degree to which children concentrate on studying, whether they check homework, or finish homework before playing. These questions were answered on a scale of 1 to 5, with higher numbers indicating greater agreement. As presented in Columns 4 to 6, robots have significantly improved children’s learning attitude. One possible side effect of intensified academic activities would be crowding out physical exercise. We examine the impact of robot adoption on children’s physical exercise frequency. The results indicate that robot adoption significantly reduces children’s weekly exercise frequency (Column 7).

**Table 6:** Effect of robots on children’s academic burden.

Variables	Log of studying time	Attending tutoring classes	Log of hours taking tutoring classes per week	Concentrate on studying	Checking homework	Finishing homework before playing	Frequency of physical exercise
	(1)	(2)	(3)	(4)	(5)	(6)	(7)
Robots	0.065*	0.082***	0.147**	0.099***	0.392***	0.078*	−0.399**
	(0.038)	(0.031)	(0.064)	(0.037)	(0.134)	(0.046)	(0.168)
Individual characteristics	Yes	Yes	Yes	Yes	Yes	Yes	Yes
City characteristics	Yes	Yes	Yes	Yes	Yes	Yes	Yes
City fixed effects	Yes	Yes	Yes	Yes	Yes	Yes	Yes
Year fixed effects	Yes	Yes	Yes	Yes	Yes	Yes	Yes
N	4,759	6,400	6,423	5,272	4,284	5,268	4,857
R^2^	0.062	0.062	0.043	0.013	0.026	0.026	0.014
Kleibergen-Paap F statistic	2162.886	2381.144	2384.449	2487.287	410.699	2475.609	2145.322

Each column represents an independent 2SLS regression. Standard errors in parentheses are clustered at the city level. ***, **, and * mean significance at the 1, 5, and 10% levels, respectively.

Academic burden is proven to be critical to children’s mental health (Kandola et al., 2020; Jiang et al., 2021). Specifically, a higher level of academic burden is associated with a higher level of school burnout, which in return, leads to a higher level of depression (Jiang et al., 2021). Furthermore, increased sitting time and reduced exercise time are linked to a higher risk of depression (Kandola et al., 2020). Our results confirm that one of the important mechanisms linking robot adoption to children’s mental health is the increased academic burden.

### 4.2 Living standards

Negative income shocks resulting from robot adoption may lower children’s living standards, thereby worsening their mental health (Lauritzen and Sivertsen, 2012; Baird et al., 2013). Firstly, we consider the living environment of children. The negative income shock caused by robots may worsen children’s living conditions, potentially leading to mental health issues (Lauritzen and Sivertsen, 2012). Column 1 of Table 7 uses a dummy variable to indicate whether the family’s living condition is poor, assigning a value of 1 if the family faces living difficulties and 0 otherwise. Living difficulties are characterized by several conditions, including: children over the age of 12 sharing a room with their parents; three generations of family members living in the same room; children of different genders over the age of 12 sharing a room; beds being laid out at night and folded up during the day; beds being placed in the living room; and other related issues. Column 2 also evaluates the impact of robot adoption on household-reported housing crowdedness, which is measured on a scale from 1 to 7, where higher values indicate greater crowding.

**Table 7:** Effect of robots on children’s living standards.

Variables	Poor living conditions	Housing crowding level	Has pocket money	Ln(Monthly pocket money)
	(1)	(2)	(3)	(4)
Robots	0.221	0.232	0.002	−0.115
	(0.292)	(0.365)	(0.035)	(0.149)
Individual characteristics	Yes	Yes	Yes	Yes
City characteristics	Yes	Yes	Yes	Yes
City fixed effects	Yes	Yes	Yes	Yes
Year fixed effects	Yes	Yes	Yes	Yes
N	3,153	4,303	6,421	6,309
R^2^	0.024	0.021	0.029	0.103
Kleibergen-Paap F statistic	258.920	422.780	2384.404	2329.375

Each column represents an independent 2SLS regression. Standard errors in parentheses are clustered at the city level. ***, **, and * mean significance at the 1, 5, and 10% levels, respectively.

Moreover, consumption is a useful indicator of living standards. However, the CFPS only reports overall household consumption and lacks data on consumption by children. Therefore, we examine the impact of robots on whether children have pocket money and the amount of their monthly pocket money (log-transformed). The pocket money allowances of children depend largely on the parents’ budget, and have been proven to significantly affect children’s mental health (Baird et al., 2013).

Columns 1 to 4 of Table 7 indicate that robots exert no significant impact on children’s living conditions or pocket money. These results suggest that robots have little influence on children’s living standards. One possible explanation is the substantial savings and assets of Chinese households, which help maintain living standards during sudden financial shortfalls. According to CFPS 2020 data, the average annual household consumption is 49,575 yuan, while the average household cash and deposits are 76,519 yuan, and the average household net assets are 807,786 yuan. Without reducing family living quality, household cash and deposits can cover 1.5 years of household consumption, and household net assets can cover 16.3 years of household consumption. Another explanation lies in the role of social safety nets, which support individuals negatively affected by shocks or emergencies. Social safety nets are commonly categorized as formal or informal, depending on whether they are legally mandated (Paitoonpong et al., 2008). In China, formal social safety nets include Dibao (the minimum living standard guarantee program), unemployment insurance and other statutory schemes, while informal social safety nets involve transfers from family members, friends, neighbors, communities, and other sources such as NGOs. Both systems play a crucial role in sustaining living standards when household income is disrupted by automation. Consequently, the short-term labor market impact of robots has not significantly undermined children’s living standards.

### 4.3 Parent–child interactions

The income difficulties caused by robots may cause parents to care less about their children’s lives and reduce positive parent–child interactions (Brody et al., 1994), which could exacerbate children’s stress responses and harm their mental health (McLoyd, 1990).

We investigate this mechanism using survey questions from the CFPS related to parent–child relationships in Table 8. In Column 1, the dependent variable is the frequency of heart-to-heart talks with parents over the past month answered by children. Columns 2 to 4 examine more detailed questions to assess the impact of robots on parent–child interactions. Three questions are used, answered by parents or children: parents take the initiative to actively communicate with the child, parents or guardians ask about what happened to the child at school, and parents or guardians praise the child. Responses to these questions are rated on a scale of 1 to 5, where 1 = Never, 2 = Occasionally, 3 = Sometimes, 4 = Often, and 5 = Always. The results from Columns 1 to 4 show that robot adoption reduces both the frequency of positive parent–child interactions and parents’ care for their children’s lives.

**Table 8:** Effect of robots on parent–child interactions.

Variables	Frequency of heart-to-heart talks with parents	Parents take the initiative to actively communicate with the child	Parents ask about what happened to the child at school	Parents praise the child	Frequency of quarrels between children and parents
	(1)	(2)	(3)	(4)	(5)
Robots	−0.305**	−0.276*	−0.104*	−0.145**	0.012
	(0.132)	(0.158)	(0.059)	(0.056)	(0.118)
Individual characteristics	Yes	Yes	Yes	Yes	Yes
City characteristics	Yes	Yes	Yes	Yes	Yes
City fixed effects	Yes	Yes	Yes	Yes	Yes
Year fixed effects	Yes	Yes	Yes	Yes	Yes
N	6,362	4,998	2,643	2,645	6,382
R^2^	0.014	0.026	0.030	0.024	0.005
Kleibergen-Paap F statistic	1969.031	264.733	3685.491	3683.190	2541.424

Each column represents an independent 2SLS regression. Standard errors in parentheses are clustered at the city level. ***, **, and * mean significance at the 1, 5, and 10% levels, respectively.

Additionally, Column 5 examines the impact of robot adoption on the negative interactions between parents and children. The frequency of quarrels with parents in the past month answered by children is used as the dependent variable. The results show that robot adoption has no significant effect on the frequency of quarrels between parents and children. These tests suggest that reduced positive interactions between parents and children also serves as a channel through which the introduction of robots contributes to children’s depression.

## 5 Heterogeneity analysis

### 5.1 Heterogeneity by gender

It has long been established that women and men respond differently to adverse shocks (Hua et al., 2023). Therefore, girls and boys may exhibit distinct psychological responses to the introduction of robots. Columns 1–2 of Table 9 show that robots indeed contribute to severe depression in girls but not boys. Investigating the source of this gender difference reveals that robots have increased the academic burden and reduced parent–child interactions for both genders (Table A1).^1^ As girls generally display a higher vulnerability in terms of mental conditions (Kuehner, 2017), robot adoption would contribute to a greater deterioration of girls’ mental conditions.

**Table 9:** Heterogeneity by children’s gender and residential areas.

	Severely depressed			
	(1)	(2)	(3)	(4)
	Girls	Boys	Urban areas	Rural areas
Robots	0.023***	0.014	0.014*	0.009
	(0.009)	(0.012)	(0.008)	(0.021)
Individual characteristics	Yes	Yes	Yes	Yes
City characteristics	Yes	Yes	Yes	Yes
City fixed effects	Yes	Yes	Yes	Yes
Year fixed effects	Yes	Yes	Yes	Yes
N	3,030	3,393	2,697	3,722
R^2^	0.010	0.005	0.011	0.005
Kleibergen-Paap F statistic	2983.142	1169.410	1517.169	837.360

Each column represents an independent 2SLS regression. Standard errors in parentheses are clustered at the city level. ***, **, and * mean significance at the 1, 5, and 10% levels, respectively.

### 5.2 Heterogeneity by residential areas

Given the varying importance that families place on education in rural and urban areas (Li et al., 2017), we would expect a different effect of robots between those two areas. As Columns 3–4 of Table 9 imply the effects of robots on children’s mental health are indeed significant in urban areas but not in rural areas. This is corroborated by the fact that urban children’s studying time has increased significantly in response to robot adoption (as shown in Column 1 of Panel A, Table A2). In contrast, robots have only increased the amount of time rural children spend in tutoring classes (see Columns 2–3 of Panel B, Table A2), without affecting their overall learning time (see Column 1 of Panel B, Table A2). This result may arise because urban parents, in response to advancements in robotics, are more likely to enhance their children’s competitiveness by extending study time. In contrast, rural parents may have limited educational awareness, focusing primarily on supervising tutoring sessions and paying little attention to extending learning time after training. The increased study time for urban children has heightened academic burden, leading to a significant deterioration in their mental health.

## 6 Conclusions and policy implications

The rapid advancement of automation may reshape various aspects of our lives. This paper finds that robot adoption can raise the likelihood of severe depression among Chinese children. By examining various mechanisms, we find that increased academic burden and reduced positive parent–child interactions play predominant roles in transmitting the effect of robots to children’s mental health. Specifically, facing higher robot adoption, children’s study time is prolonged, their likelihood of attending tutoring classes increases, and their exercise time decreases, which takes a toll on their mental health. The research further tests whether robots harm children’s mental health through negative family income shocks and finds that while robots do not significantly affect children’s living standards, they notably reduce positive parent–child interactions, which could exacerbate children’s stress responses and harm their mental health. Moreover, the effects are concentrated on girls and children living in urban areas.

This paper extends the growing body of research on the impact of robots on individual well-being. It broadens the understanding of the social implications of automation, particularly for younger, more vulnerable populations. Our study affirms the existence of a “hidden cost of automation” in society. The impacts of robots are not restricted to the current workforce, but also affect the mental health of future generations. Our findings emphasize the importance of addressing social security concerns and enhancing social assistance nets to offset the concealed costs imposed by robots on children.

The results have some important policy implications. Given the potential social and private costs of children’s depression, our research calls for increased governmental attention to the psychological effects of automation on children. First, it is crucial to mitigate the negative impact of robots on the labor market and protect job opportunities for the current workforce, thus reducing the impact of robots on the next generation. Educational and training programs are necessary to help people be better prepared to face and overcome automation. There is a special need to intensify skills training for low-skilled individuals, who have been significantly impacted by robots. Second, reducing children’s excessive academic pressure and promoting physical activity are essential to protecting their mental health. The government could limit both the amount of homework assigned to children and the duration of after-school tutoring, thus reducing their academic burden and allowing more time for leisure and physical activity. Third, our findings emphasize the pivotal role of parent–child interactions. Policies should be designed to help normalize supportive and meaningful parent–child relationships, thereby reducing the risk of childhood depression. Schools and community centers could offer workshops and parenting courses that promote active listening, empathy, and constructive communication through activities such as parent–child discussions and problem-solving games. Public campaigns could also encourage families to dedicate regular time to shared meals or outdoor activities without digital distractions. Fourth, the government and schools should collaborate to enhance care for children’s mental health. The government could allocate sufficient funding for training more qualified mental health professionals for adolescents, while schools could increase the availability of mental health professionals to help children solve psychological issues promptly.

This study has several limitations and possible extensions for further research. First, while the shift-share design provides a credible way to translate industry-level robot data into city-level robot adoption, it inevitably introduces potential measurement concerns. This approach assumes that a city’s robot exposure is proportional to its pre-determined industry employment structure. If actual city-level adoption deviates from this proportionality assumption, the measure may introduce attenuation bias, which would likely result in a conservative estimate of the true effect size. Therefore, the reported magnitudes should be interpreted with caution. Future research should employ more precise measures of actual robot adoption to better capture city-level penetration of industrial robots and provide more accurate estimates. Second, due to data limitations, this article only examines the short-term impact of robots on children’s mental health. Further research could track the long-term impacts of automation on children’s human capital, mental health, educational development, emotional development, and eventual income levels in adulthood. Third, this paper primarily focuses on the impact of industrial robots, which capture only one dimension of automation. Besides industrial robots, the rapid development of service robots may also impact children’s mental health. This aspect also warrants further study. Fourth, this study focuses solely on China. Cross-country comparisons in other developing nations would be a valuable direction for future research, which could help assess the generalizability of the findings.

## Data Availability

The raw data supporting the conclusions of this article will be made available by the authors, without undue reservation.

## Footnotes

^1^Further analysis shows that robot adoption does not have a significant differential impact on parental expectations or educational investments between boys and girls.
