# Supplementary material for: The hidden costs of automation: does robot adoption affect children’s mental health?
Source: Front Psychol. 2025 Oct 9;16:1643849. doi: 10.3389/fpsyg.2025.1643849 (PMC12546223; doi:10.3389/fpsyg.2025.1643849)
Supplement: Supplementary file 1 [file Table_1.DOCX]

Supplementary Material

**Table A1** Effect of robots on children’s academic burden and parent-child interactions for girls and boys

|  | Children’s academic burden | | | | | | | Parent-child interactions | | | | |
| --- | --- | --- | --- | --- | --- | --- | --- | --- | --- | --- | --- | --- |
| **Variables** | Log of studying time | Attend tutoring classes | Log of hours taking tutoring classes per week | Concentrate on studying | Check homework | Finish homework before playing | Frequency of physical exercise | Frequency of heart-to-heart talk with parents | Parents take the initiative to actively communicate with the child | Parents ask about what happened to the child at school | Parents praise the child | Frequency of quarrels between children and parents |
|  | (1) | (2) | (3) | (4) | (5) | (6) | (7) | (8) | (9) | (10) | (11) | (12) |
| ***Panel A: Girls*** |  |  |  |  |  |  |  |  |  |  |  |  |
| Robots | 0.067* | 0.085*** | 0.165*** | 0.070 | 0.255 | 0.047 | -0.399 | -0.374** | -0.245 | 0.023 | -0.067 | -0.226 |
|  | (0.036) | (0.028) | (0.051) | (0.061) | (0.228) | (0.047) | (0.243) | (0.188) | (0.190) | (0.087) | (0.069) | (0.180) |
| Observations | 2,229 | 3,019 | 3,030 | 2,496 | 2,027 | 2,495 | 2,279 | 2,995 | 2,355 | 1,223 | 1,224 | 3,012 |
| R-squared | 0.088 | 0.055 | 0.040 | 0.010 | 0.020 | 0.018 | 0.007 | 0.012 | 0.026 | 0.044 | 0.036 | 0.008 |
| Kleibergen-Paap  F statistic | 3316.456 | 2972.145 | 2982.151 | 1918.409 | 297.304 | 1894.747 | 2740.888 | 2139.499 | 240.975 | 4908.975 | 4904.379 | 3306.619 |
| ***Panel B: Boys*** |  |  |  |  |  |  |  |  |  |  |  |  |
| Robots | 0.105** | 0.094* | 0.152 | 0.166*** | 0.482*** | 0.138** | -0.569** | -0.200 | -0.305* | -0.230*** | -0.215** | 0.265 |
|  | (0.049) | (0.050) | (0.099) | (0.060) | (0.176) | (0.057) | (0.274) | (0.160) | (0.159) | (0.084) | (0.083) | (0.233) |
| Observations | 2,528 | 3,381 | 3,393 | 2,776 | 2,257 | 2,773 | 2,576 | 3,367 | 2,643 | 1,416 | 1,417 | 3,370 |
| R-squared | 0.042 | 0.065 | 0.044 | 0.010 | 0.021 | 0.020 | 0.017 | 0.017 | 0.034 | 0.028 | 0.025 | 0.007 |
| Kleibergen-Paap  F statistic | 1140.470 | 1165.331 | 1169.064 | 1842.463 | 555.453 | 1860.785 | 1136.761 | 1134.437 | 255.663 | 1925.183 | 1923.201 | 1183.911 |

**Notes**: Each column in each panel represents an independent 2SLS regression. All regressions include individual and city characteristics, and city and year fixed effects. Standard errors in parentheses are clustered at the city level. ***, **, and * mean significance at the 1%, 5%, and 10% levels, respectively.

**Table A2** Effect of robots on children’s academic burden and parent-child interactions in urban and rural areas

|  | Children’s academic burden | | | | | | | Parent-child interactions | | | | |
| --- | --- | --- | --- | --- | --- | --- | --- | --- | --- | --- | --- | --- |
| **Variables** | Log of studying time | Attend tutoring classes | Log of hours taking tutoring classes per week | Concentrate on studying | Check homework | Finish homework before playing | Frequency of physical exercise | Frequency of heart-to-heart talks with parents | Parents take the initiative to actively communicate with the child | Parents ask about what happened to the child at school | Parents praise the child | Frequency of quarrels between children and parents |
|  | (1) | (2) | (3) | (4) | (5) | (6) | (7) | (8) | (9) | (10) | (11) | (12) |
| ***Panel A: Urban area*** | |  |  |  |  |  |  |  |  |  |  |  |
| Robots | 0.134*** | 0.061* | 0.144** | 0.071 | 0.406** | 0.039 | -0.271 | -0.084 | -0.078 | -0.184** | -0.102 | 0.096 |
|  | (0.043) | (0.033) | (0.070) | (0.046) | (0.202) | (0.053) | (0.221) | (0.171) | (0.171) | (0.079) | (0.070) | (0.173) |
| Observations | 2,045 | 2,690 | 2,697 | 2,199 | 1,767 | 2,198 | 2,080 | 2,673 | 2,061 | 1,076 | 1,076 | 2,674 |
| R-squared | 0.086 | 0.051 | 0.032 | 0.017 | 0.032 | 0.028 | 0.016 | 0.022 | 0.029 | 0.045 | 0.037 | 0.008 |
| Kleibergen-Paap  F statistic | 1283.846 | 1486.748 | 1516.602 | 1779.499 | 397.181 | 1778.864 | 1224.662 | 1492.662 | 190.399 | 2534.569 | 2534.569 | 1621.124 |
| ***Panel B: Rural area*** |  |  |  |  |  |  |  |  |  |  |  |  |
| Robots | -0.046 | 0.127*** | 0.237** | 0.132 | 0.385 | 0.184 | -0.196 | -0.178 | -0.465** | -0.019 | -0.010 | -0.167 |
|  | (0.058) | (0.046) | (0.105) | (0.091) | (0.492) | (0.126) | (0.400) | (0.338) | (0.181) | (0.122) | (0.100) | (0.476) |
| Observations | 2,709 | 3,706 | 3,722 | 3,068 | 2,509 | 3,065 | 2,772 | 3,685 | 2,930 | 1,557 | 1,559 | 3,704 |
| R-squared | 0.054 | 0.034 | 0.024 | 0.013 | 0.018 | 0.028 | 0.011 | 0.015 | 0.017 | 0.035 | 0.021 | 0.005 |
| Kleibergen-Paap  F statistic | 768.541 | 845.767 | 837.134 | 489.026 | 935.758 | 482.110 | 744.947 | 568.930 | 653.566 | 1106.218 | 1105.685 | 840.176 |

**Notes**: Each column in each panel represents an independent 2SLS regression. All regressions include individual and city characteristics, and city and year fixed effects. Standard errors in parentheses are clustered at the city level. ***, **, and * mean significance at the 1%, 5%, and 10% levels, respectively.
